# Motion Similarity Evaluation between Human and a Tri-Co Robot during Real-Time Imitation with a Trajectory Dynamic Time Warping Model

**DOI:** 10.3390/s22051968

**Published:** 2022-03-02

**Authors:** Liang Gong, Binhao Chen, Wenbin Xu, Chengliang Liu, Xudong Li, Zelin Zhao, Lujie Zhao

**Affiliations:** School of Mechanical Engineering, Shanghai Jiao Tong University, Shanghai 200240, China; cbh_mage@sjtu.edu.cn (B.C.); wenbinx@princeton.edu (W.X.); chlliu@sjtu.edu.cn (C.L.); lxd834740904@alumni.sjtu.edu.cn (X.L.); sjtuytc@sjtu.edu.cn (Z.Z.); zhaolujie@alumni.sjtu.edu.cn (L.Z.)

**Keywords:** motion imitation, life-size humanoid robot, BioVision hierarchy, motion capture, DTW-based trajectory evaluation, human-in-the-loop control

## Abstract

Precisely imitating human motions in real-time poses a challenge for the robots due to difference in their physical structures. This paper proposes a human–computer interaction method for remotely manipulating life-size humanoid robots with a new metrics for evaluating motion similarity. First, we establish a motion capture system to acquire the operator’s motion data and retarget it to the standard bone model. Secondly, we develop a fast mapping algorithm, by mapping the BVH (BioVision Hierarchy) data collected by the motion capture system to each joint motion angle of the robot to realize the imitated motion control of the humanoid robot. Thirdly, a DTW (Dynamic Time Warping)-based trajectory evaluation method is proposed to quantitatively evaluate the difference between robot trajectory and human motion, and meanwhile, visualization terminals render it more convenient to make comparisons between two different but simultaneous motion systems. We design a complex gesture simulation experiment to verify the feasibility and real-time performance of the control method. The proposed human-in-the-loop imitation control method addresses a prominent non-isostructural retargeting problem between human and robot, enhances robot interaction capability in a more natural way, and improves robot adaptability to uncertain and dynamic environments.

## 1. Introduction

In 2017, the National Natural Science Foundation of China (NSFC) launched a major research project, the Tri-Co Robot (Coexisting-Cooperative-Cognitive Robot). Tri-Co Robots are those that can naturally interact and collaborate with the operating environment, humans, as well as other robots and that are adaptive to complex dynamic environments [1]. Over the years, Tri-Co robots have formed many different types due to different scenarios, different functions, and different tasks completed, where human–robot interaction (HRI) has become an important research field and has received extensive attention in academia and industry.

The working scenes of robots are mainly scenes of human life, and most of these scenes are constructed according to human scales, needs, and capabilities. Whether it is industrial robots, agricultural robots, or various service robots, humanoid robots have relative advantages when replacing or helping humans in their work. Compared to the general-purpose HRIs, humanoid robots have many advantages. First of all, humanoid robots have the same structure and scale as humans, which means that they can imitate most of the actions that humans can do. Secondly, humanoid robots provide a platform for the subsequent development of HRIs. Due to the similar structures between humanoid robots and humans, human experience can give first-person guidance to robots in the form of teaching and can even derive humanoid autonomous decision-making methods. Thirdly, humanoid robots can use existing human knowledge and skills to improve performance and greatly reduce the cost of HRI. Our team has proposed a kind of human robot control method [2], which is used to control humanoid robots so that the robots can imitate human motion.

It is a valuable method to teach robots behaviors that are not pre-programmed naturally, and it promotes the interactivity between humans and humanoid robots. Taking humans as an example, humans always learn new knowledge and skills through imitation [3]. For humanoid robots, it is usually easier to imitate human behavior than to program the controller directly [4]. Therefore, it is particularly important for humanoid robots to imitate humans. Humanoid behavior is the basis of humanoid robot motion [5].

There are several related works over the past few years. Marcia Riley et al. use an external camera and the operator’s head-mounted camera to obtain body posture, and calculated the joint angle by a fast full-body inverse kinematics (IK) method. They use this method to realize a real-time simulation of a Sarcos humanoid robot with 30 degrees of freedom (DOF). In addition, some articles ([1,6,7,8]) use Kinect to collect pictures, perform gesture recognition, and reproduce similarities on humanoid robots through various algorithms actions. With the upgrading of machine vision algorithms, some new methods have emerged. Emily-Jane Rolley-Parnell uses an RGB-D camera to photograph human movements, obtains image information and depth information from it, uses an openpose algorithm to obtain two-dimensional information of human skeleton posture, and realizes the control of humanoid robots through solution [9]. In addition to vision, researchers have also tried other sensory methods. Abhay Bindal fixes an accelerometer motion sensor and an infrared sensor on the human leg to obtain data to control the gait movement of biped robots in real time [10]. Akif DURDU connects potentiometers to human joints, and after classification by neural network, controls the robot to perform movement [4]. Shingo Kitagawa uses a newer method: they develop a miniature tangible cube. They use this cube to obtain the controller’s arm information, thus realizing the control of the robot’s arms [11]. However, there are some demerits in existing works. First of all, the use of vision for motion recognition and control is not reliable because these methods are sensitive to lighting conditions and complex backgrounds. With wearable sensors, the accuracy of motion control will be significantly improved. Secondly, solving the whole-body IK problem will inevitably affect the real-time performance. Thirdly, motion imitation has rarely been applied to humanoid robots with human-level dexterity due to the pending issue of how to deal with the incongruent geometrics between humans and robots.

In this article, we propose a human-in-the-loop system, which implements the robot imitating the real-time motion of human upper limbs on the life-size open source 3D humanoid robot InMoov. The InMoov robot has a structure similar to human beings, with a total of 29 degrees of freedom, 22 of which are used and controlled in our system. The flow of motion simulation is as follows. First, we use wearable sensors to capture the movement of the upper limbs of the human body. These data are saved in the BVH (BioVision Hierarchy) format and transmitted to the robot controller industrial computer. Next, the BVH data are analyzed by mathematical methods and converted into the corresponding joint angle data of the robot. Finally, the industrial computer sends the joint angle data to the lower controller in real time to control the robot. This kind of human-in-the-loop system provides a novel, real-time, and accurate method for the imitation of human actions on humanoid robots. In addition, this paper also proposes a trajectory evaluation method, which is based on DTW (Dynamic Time Warping), to evaluate the similarity between human behavior and robot motion.

This paper is organized as follows. In Section 2, the motion capture system is introduced. Section 3 discusses the setup of the humanoid robot. Section 4 presents the realization of real-time motion imitation on the humanoid robot, which provides a quantitative model for describing the incongruent feature between human and robot. Section 5 conducts several experiments on complicated gesture imitation through our proposed method. Finally, Section 6 and Section 7 give the discussions and conclusions.

## 2. Motion Capture System

This section introduces the motion capture system. The system mainly includes a motion sensor for capturing human motion and a human motion redirection method that links human motion with a simplified skeleton model.

### 2.1. Motion Sensor

We used a wearable sensor designed by Noitom Technology Ltd. in Beijing, China. It is a system composed of 32 9-axis sensors. This system is small in size, easy to wear, and has strong applicability. By connecting with Axis Neuron Pro (ANP) on the Windows operating system, the system can perform calibration and data transmission management. At the same time, the collected data can visually reflect the operator’s movement in ANP.

### 2.2. Human Motion Retargeting

Motion redirection is a classic problem, which aims to redistribute and combine the motion of one object to another while keeping the two motion styles consistent [12]. By using motion redirection, BVH data can be used to reproduce the human motion collected by the sensor on the ANP bone model. BVH data can store the hierarchical movement of the skeleton, that is, the movement of the child node depends on the movement of the parent node [13].

The BVH data we use do not include position channels; each joint uses only three rotation data while keeping the length of the bones connecting the joints unchanged. Next, since the wearable sensor can be regarded as being fixed on the operator, the posture of the operator can be calculated through the three rotation angles of each joint.

## 3. Setup of Humanoid Robot

The humanoid robot has a humanoid design, which has a similar structure and scale to the human body, and can imitate the movement of the human body [14]. However, due to the complexity of the human body structure and the limitations of traditional manufacturing methods, there have been few subtle humanoid robot designs for a long time. Today, with the rapid development of 3D printing technology, 3D-printed humanoid robots such as InMoov, Flobi, and iCub are designed to be used as experimental platforms for HRI research.

**Figure 1:**
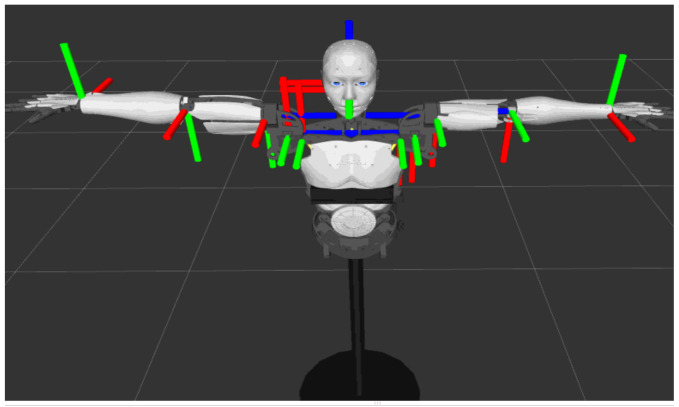
DOF of the humanoid robot (DOF of fingers are not shown).

The research in this article is based on the InMoov 3D printed life-size humanoid robot initiated by French sculptor Gael Langevin in 2012 [15]. The InMoov robot contains a total of 29 degrees of freedom, 22 of which are controlled in the motion simulation of this article, including 5 DOF for each hand, 4 for each arm, 3 for each shoulder, and 2 for the neck, as shown in Figure 1. In terms of control, the upper controller uses the Arduino Mega 2560. On the one hand, the upper controller needs to communicate with the industrial computer to retrieve the joint angle control information. On the other hand, it needs to communicate with the lower controllers, which include four Arduino Nano boards, through the Modbus RTU protocol. Each Arduino Nano controls the movement of six servos through PWM.

## 4. Real-Time Imitation of Human Motion

**Figure 2:**
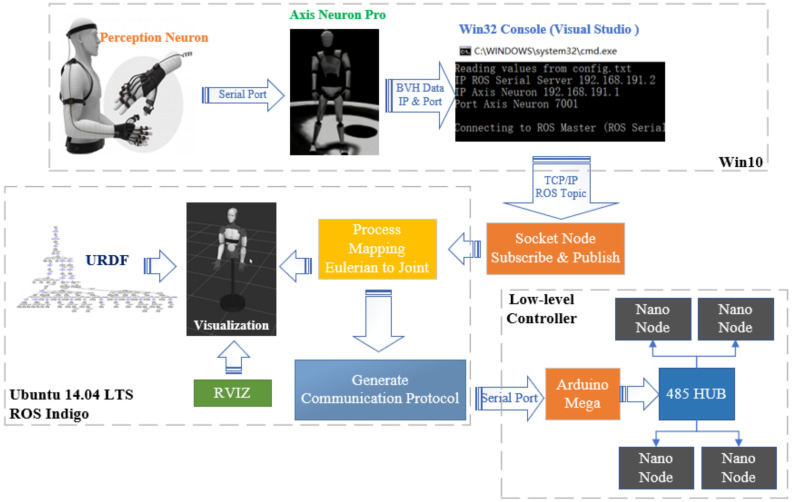
Whole structure of the proposed method.

The overall structure of the proposed method is shown in Figure 2. First, the ROS (Robot Operating System) operating system is adopted to use message subscription and publication for data communication to ensure the security of data transmission. Secondly, a fast mapping algorithm is established to convert the Euler angle of each joint in the BVH data into the joint angle of the corresponding joint in a very simplified way. Thirdly, the trajectory evaluation function is used to quantify the degree of similarity between the trajectory of the robot and the trajectory of humans. Fourthly, the collected human movement and robot movement can be observed and compared on different visualization terminals.

### 4.1. Data Transmission

**Figure 3:**
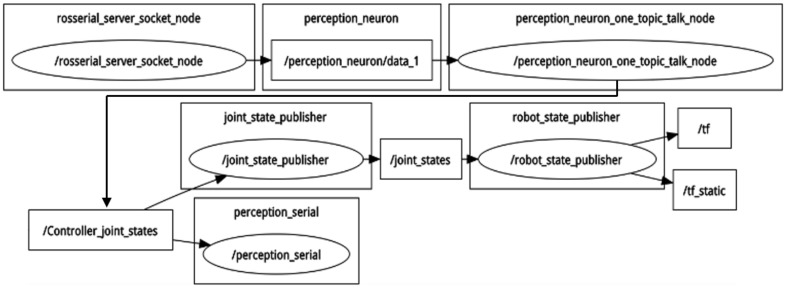
Visualized data stream through ROS publish_subscribe messaging.

Nodes, which are the message processing units in the ROS system, are used to subscribe or publish messages to ROS topics [16]. The data flow of the system in this article is visualized in Figure 3, where ellipses represent nodes and squares represent topics.

- rosserial_server_socket_node connects with the win32 console through TCP/IP and then advertises the topic, perception_neuron/data_1;
- perception_neuron_one_topic_talk_node subscribes to the previous topic and then converts Euler angles in BVH data to joint angles, which are then published to another topic called Controller_joint_states;
- joint_state_publisher subscribes to the previous topic and realizes the real-time simulation of robot model;
- perception_serial will send joint angles to the low-level slave controller through a serial port after obtaining them from Contoller_joint_states.

**Figure 4:**
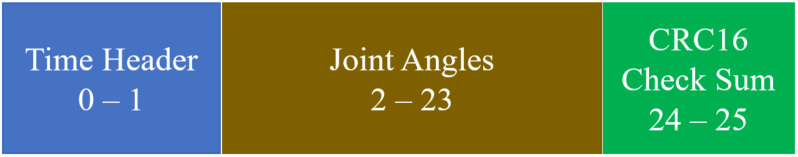
Designed Communication Protocol.

The above content shows that the data transmission on the industrial computer is mainly carried out on ROS. After the data leave the industrial computer, the packaged joint angle needs to be transmitted to the upper and lower controllers through the serial port. In order to prevent packet loss or data misalignment during transmission, we designed a specific communication protocol, as shown in Figure 4. The time stamp data and joint angle data are converted into integers through a specific encoding method in the protocol. The communication protocol includes 2 bits of time stamp data, 22 bits of position data corresponding to each joint, and 2 bits of CRC16 check code, which are generated based on the first 27 bits to ensure the safety of data transmission.

### 4.2. Mapping Algorithm

In order for the InMoov robot to imitate the motion of the human body, it is necessary to design an algorithm to calculate the corresponding joint angle based on the Euler angle in the BVH data. Through the three Euler angles of each joint in BVH, we can calculate the rotation matrix between the child link and the parent link. Assuming that the Euler angle of rotation order ZYX can be expressed as φ,θ,ψ, the rotation matrix of the child frame relative to the parent frame is:

(1) $$R_{child}^{parent}=\;\left(\begin{array}{ccc} cos\varphi & -sin\varphi & 0\\ sin\varphi & cos\varphi & 0\\ 0 & 0 & 1 \end{array}\right)\left(\begin{array}{ccc} cos\theta & 0 & sin\theta \\ 0 & 1 & 0\\ -sin\theta & 0 & cos\theta \end{array}\right)\left(\begin{array}{ccc} 1 & 0 & 0\\ 0 & cos\psi & -sin\psi \\ 0 & sin\psi & cos\psi \end{array}\right)$$

**Figure 5:**
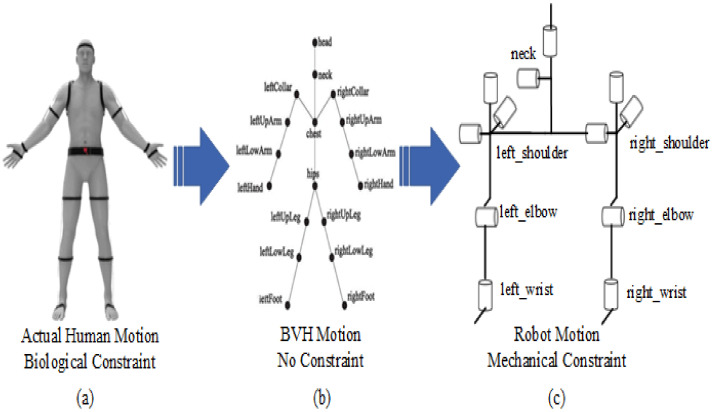
Three motion systems with different constraints. (**a**) shows the human motion system with biological constraint. (**b**) shows the BVH motion system with no constraint. (**c**) shows the robot motion system with mechanical constraint.

Figure 5 shows the mapping problem. The joints of humans and humanoid robots are not exactly the same. Limited by mechanical constraints, some joints of humanoid robots cannot achieve rotation in three independent directions. For each joint, the situation is different, so we need to formulate algorithms for different situations.

The first case is that the degrees of freedom of the human joints are the same as the degrees of freedom of the robot joints. Take the shoulders as an example. The shoulders of the InMoov robot are similar to the shoulders of the human body, both have three degrees of freedom, and their rotation axes can be approximately regarded as perpendicular to each other. Assuming the joint angles of three shoulder joints are relatively α,β,γ, the rotation matrix of the arm coordinate system relative to the shoulder coordinate system can be expressed as:

(2) $$R_{arm}^{shoulder}=\;\left(\begin{array}{ccc} cos\alpha & -sin\alpha & 0\\ sin\alpha & cos\alpha & 0\\ 0 & 0 & 1 \end{array}\right)\left(\begin{array}{ccc} cos\beta & 0 & sin\beta \\ 0 & 1 & 0\\ -sin\beta & 0 & cos\beta \end{array}\right)\left(\begin{array}{ccc} 1 & 0 & 0\\ 0 & cos\gamma & -sin\gamma \\ 0 & sin\gamma & cos\gamma \end{array}\right)$$

From the formula, you only need to make the φ,θ,ψ angle obtained from the motion sensor equal to the α,β,γ angle of the control robot. The only thing to note is that the rotation sequence of the two must be the same.

**Figure 6:**
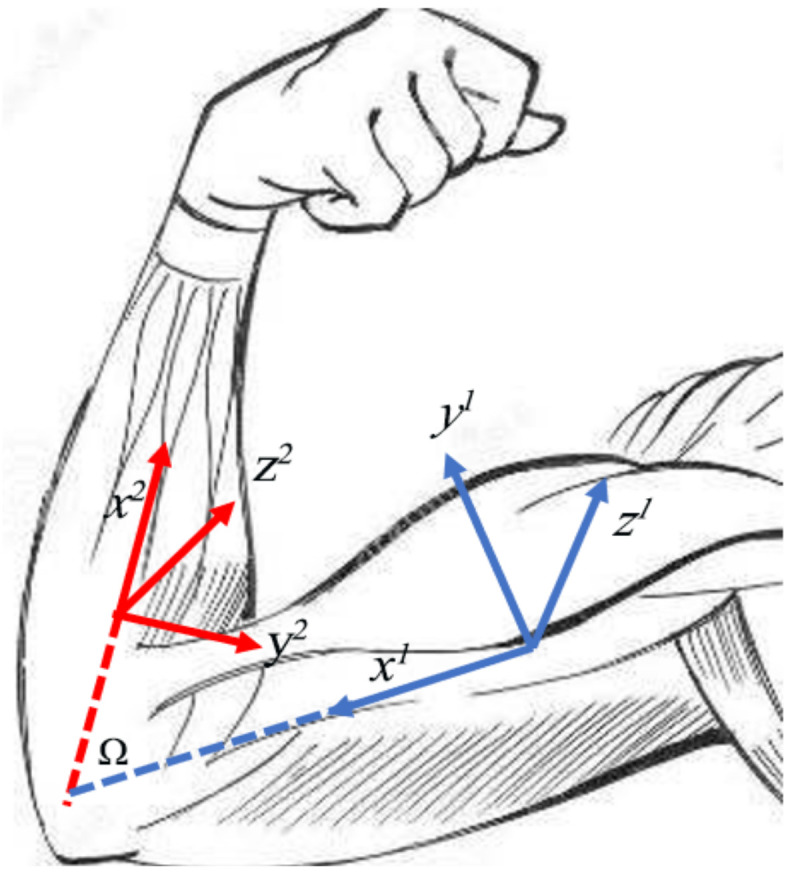
Elbow conversion from 2 DOF to 1 DOF. $x^{1}y^{1}z^{1}$ and $x^{2}y^{2}z^{2}$ are the DH coordinate systems connecting the two links of the elbow joint, respectively. Ω is the bending angle of the elbow joint.

The second one is conversion from two human DOF to one robot DOF. In the upper limbs, this type of conversion mainly includes the elbow and wrist. Take the elbow as an example. The elbow of a human can bend and rotate, while the elbow of a robot can only bend. In order to calculate the bending joint angle Ω of the elbow of the robot, as shown in the Figure 6, with the assumptions that sensors are fixed with respect to the human body and the x-direction is along the links, we can derive the following equations with the rotation matrix (1). $R_{2}^{1}$ stands for the rotation matrix of frame $x_{2}y_{2}z_{2}$ with respect to $x_{1}y_{1}z_{1}$. ${\hat{x_{1}}}^{1}$ is the description of unit vector of $x_{1}$ in frame $x_{1}y_{1}z_{1}$.

(3) $${\hat{x_{2}}}^{2}={\left(1,0,0\right)}^{T}$$

(4) $${\hat{x_{2}}}^{1}=R_{2}^{1}{\hat{x_{2}}}^{2}={\left(cos\theta cos\varphi ,cos\theta sin\varphi ,-sin\theta \right)}^{T}$$

(5) $$<{\hat{x_{2}}}^{1},{\hat{x_{1}}}^{1}>=arccos\left(cos\theta cos\varphi \right)$$

(6) $$\Omega =\pi -<{\hat{x_{2}}}^{1},{\hat{x_{1}}}^{1}>=\pi -arccos\left(cos\theta cos\varphi \right)$$

**Figure 7:**
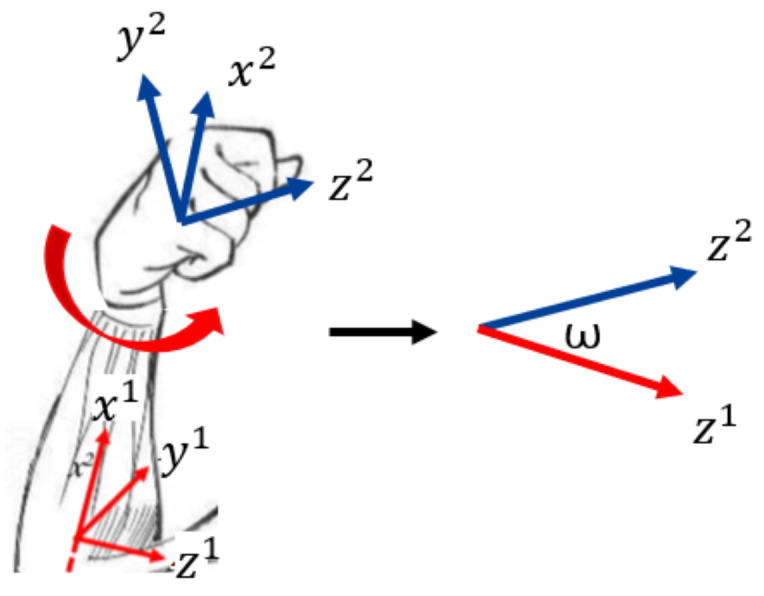
Wrist conversion from 2 DOF to 1 DOF. $x^{1}y^{1}z^{1}$ and $x^{2}y^{2}z^{2}$ are the DH coordinate systems connecting the two links of the elbow joint, respectively. ω is the rotating angle of the elbow joint.

The wrist is similar to the elbow. The difference is that the robot wrist can only rotate rather than bend. We need to compute the joint angle for rotating, which is ω, as shown in Figure 7. We apply the same mathematical notation settings above and obtain the following results:

(7) $${\hat{z_{2}}}^{2}={\left(0,0,1\right)}^{T}$$

(8) $${\hat{z_{2}}}^{1}=R_{2}^{1}{\hat{z_{2}}}^{2}=\left(\begin{array}{c} sin\varphi sin\psi +cos\varphi sin\theta cos\psi \\ -cos\varphi sin\psi +sin\varphi sin\theta sin\psi \\ cos\theta cos\psi \end{array}\right)$$

(9) $$<{\hat{z_{2}}}^{1},{\hat{z_{1}}}^{1}>=arccos\left(cos\theta cos\psi \right)$$

(10) $$\omega =<{\hat{z_{2}}}^{1},{\hat{z_{1}}}^{1}>=arccos\left(cos\theta cos\psi \right)$$

**Figure 8:**
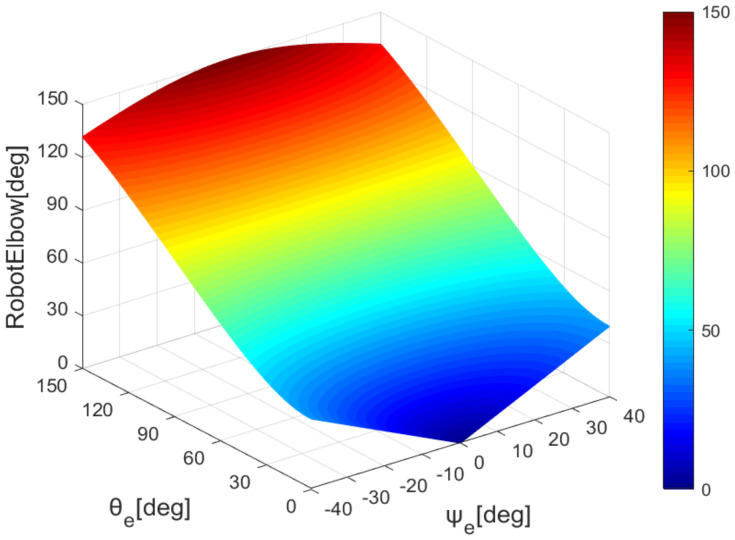
Elbow joint angle map.

**Figure 9:**
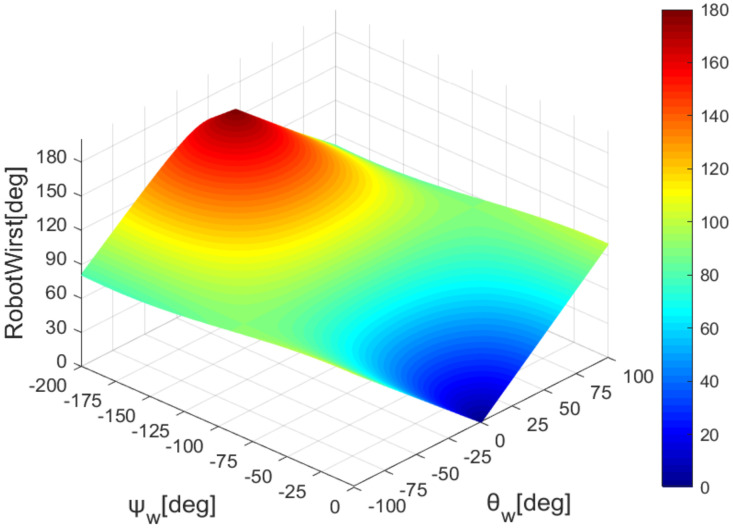
Wrist joint angle map.

According to formulas 6 and 10, we draw the mapping of the robot’s elbow and wrist with the sensor’s data, as shown in Figure 8 and Figure 9.

The last case is conversion from three human DOF to two robot DOF, such as in the neck joint. The solution to this case resembles that for the shoulder joint, and we only need to take two of three Euler angles in the corresponding order.

### 4.3. Trajectory Imitation Evaluation

**Figure 10:**
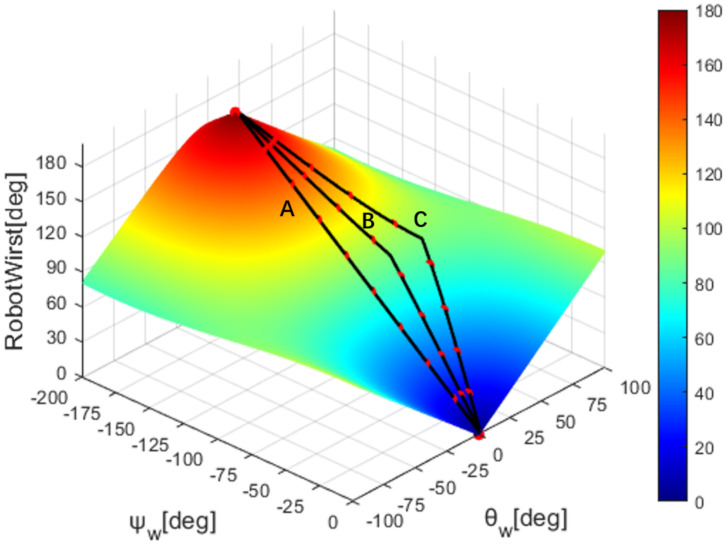
Schematic diagram of three human trajectories mapped to the same robot trajectory.

The elbow and wrist joints of robots are different from human upper limbs and lack some degrees of freedom. This leads to the fact that the robot cannot perform one-to-one correspondence of joints when imitating human actions. Robots need to map human movements to themselves through a mapping algorithm. In this case, multiple motion trajectories of the human operator may be mapped to the same robot trajectory, as shown in Figure 10, if we apply our proposed mapping algorithm. To this end, we need a method to quantitatively assess the degree of similarity between human motion trajectories and robot trajectories. So, we propose the DTW trajectory evaluation method.

Taking wrist mapping as an example, as shown in the Figure 10, three motion trajectories of human operators are drawn, marked as A, B, and C, respectively, and the robot trajectories they map to are the same. Each trace has several marker points, which are sample points for the simulation. The trajectory A is a special trajectory, which is a movement trajectory made by making the wrist bending angle $\theta_{w}=0$. From the data sheet, the robot trajectories mapped by these three trajectories are the same, from 0 degrees to 180 degrees, and the overall time length is the same, but there is a scaling phenomenon in the time series. Reaching the same robot angle, different human trajectories differ by a maximum of 20 frames. If the point-to-point error calculation is performed directly according to the time series, there will be a time deviation between the corresponding two points used for the calculation. This approach fails to capture how similar the overall trajectories are. The introduction of the DTW distance can be used to solve this timing drift phenomenon. Obviously, with the continuous advancement of the trajectory, the accumulation of point-to-point distances will only continue to increase, while the DTW distance will vary according to the overall similarity of the trajectory.

For a trajectory with *n* discrete moments, we build a DTW square matrix $D^{n\times n}$ to describe the DTW distance between human motion trajectory and robot trajectory. In this matrix, $d_{i,j}$ means the DTW distance between the human trajectory data at time *i* and the robot trajectory data at time *j*. In addition, the elements in *D* obey the following iterative relationship, where the $s_{i}$ means the human trajectory data at time *i*, and $r_{j}$ means the robot trajectory data at time *j*. For a trajectory used for evaluation, any cut at a certain moment can be regarded as an independent trajectory. Therefore, the diagonal elements of the DTW matrix *D*, that is, diag(D), can be selected for drawing, which can more intuitively and dynamically show the similarity of the trajectory in the process.

(11) $$d_{i,j}=distance\left(s_{i},r_{j}\right)+min\left(d_{i-1,j-1},d_{i-1,j},d_{i,j-1}\right)$$

where the $distance(s_{i},r_{j})$ is the Euclidean distance between $s_{i}$ and $r_{j}$.

Our method uses DTW to calculate the timing similarity of various body parts, and then we set weights for every joint to calculate the weighted average of the trajectory differences of the whole system. Take the imitation of the arm motion of a humanoid robot as an example. Suppose the robot has three degrees of freedom at the shoulder, and one degree of freedom at the elbow and wrist, so that each degree of freedom will produce a DTW distance. We take the range of activity of each degree of freedom as its respective weight. $W_{*}$ is the weight of each joint angle, $D_{*}$ is DTW distance of each joint angle, and $A_{w}$ is the DTW distance of the robot system:

(12) $$W_{x}=max\left(x\right)-min\left(x\right)$$

(13) $$A_{w}=\frac{W_{s1}D_{s1}+W_{s2}D_{s2}+W_{s3}D_{s3}+W_{e}D_{e}+W_{w}D_{w}}{W_{s1}+W_{s2}+W_{s3}+W_{e}+W_{w}}$$

### 4.4. Different Visualization Terminals

**Figure 11:**
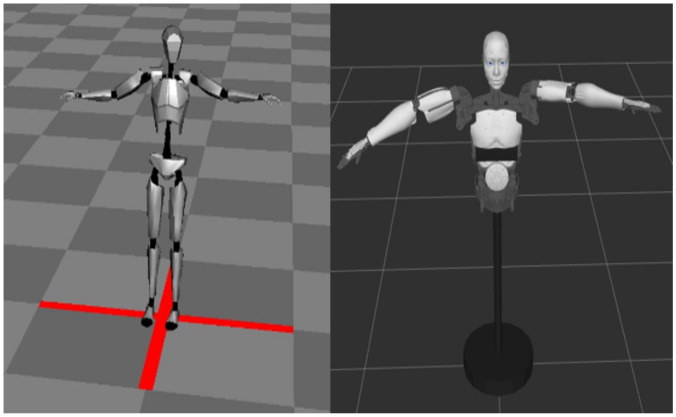
Different visualization terminals for different motion systems.

On one hand, as shown in Figure 11 on the left, ANP provides a skeletal model to visualize the human movement collected by the sensor. The skeleton model analyzes the BVH data and uses Euler angles to show the rotation of each joint of the operator.

On the other hand, as shown in Figure 11 on the right, ROS provides a simulation environment that can visualize the robot model. This requires converting the robot’s 3D model into URDF (unified robot description format) format. URDF is an XML-based language that mainly describes the general robot simulation model in the ROS system, including the shape, size, color, kinematics, and dynamic characteristics of the model [16]. We import the open source robot STL file into URDF after adjusting the scale. Then, we use Xacro (XML Macros) to reuse a structure for two different parts, namely, the left arm and the right arm, and automatically generate a URDF file. The Table A1 shows some basic syntax. Finally, we call RVIZ (a visualization tool in ROS) to visualize the robot model and make it run in real time according to the calculated joint angle.

## 5. Results

**Figure 12:**
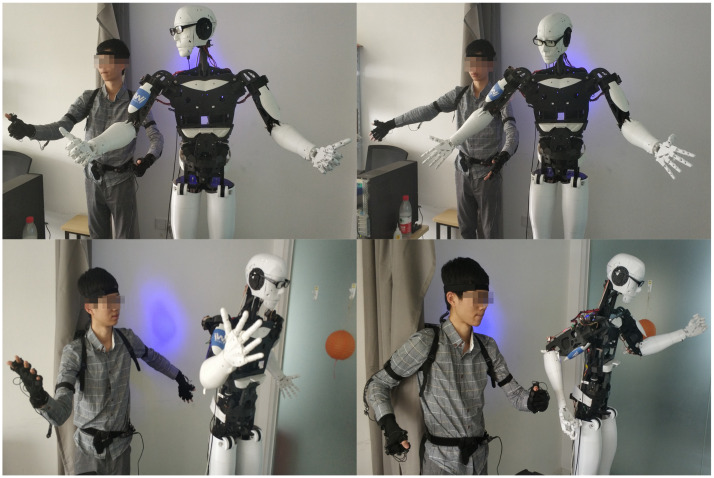
Experiments of different gestures with arms and head.

**Figure 13:**
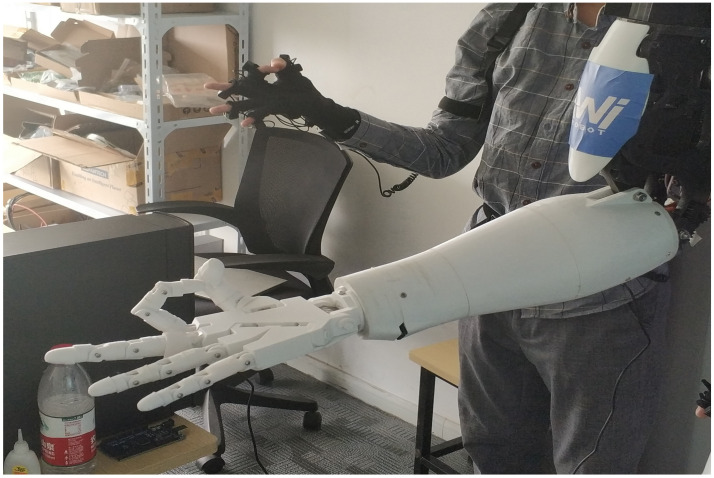
Comparison between fingers.

This section presents the experimental results using the proposed method based on the humanoid robot. The results can be seen from Figure 12 and Figure 13. To verify the feasibility of the system, we take various photos from the human motion imitation system, including different positions of two arms, face orientations and movements of fingers. These gestures are complicated because imitation of these gestures entails the rotation of most revolute joints at the same time rather than one or two. In addition, the consistency between the wearer’s action and the humanoid robot’s action has demonstrated that the robot has successfully followed the motion of the wearer’s upper limbs, thus proving the feasibility of our proposed method. In addition, the synchronous latency of less than 0.5 seconds validates the real-time performance.

**Figure 14:**
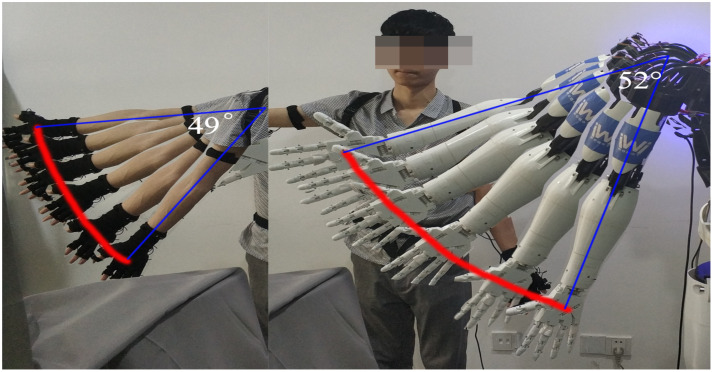
Snapshots for motion trajectory.

We use a degree of freedom rotation experiment on the right shoulder to illustrate the accuracy of our system. Figure 14 shows the comparison of the trajectory of the operator and the humanoid robot. The operator makes an arc trajectory, and his arm rotates 49°, while the humanoid robot turns over 52° under real-time control, the absolute error is close to 3°, which is about 6.1% of the rotation angle of the human arm. The relative error is small, which proves that our method has high accuracy.

**Figure 15:**
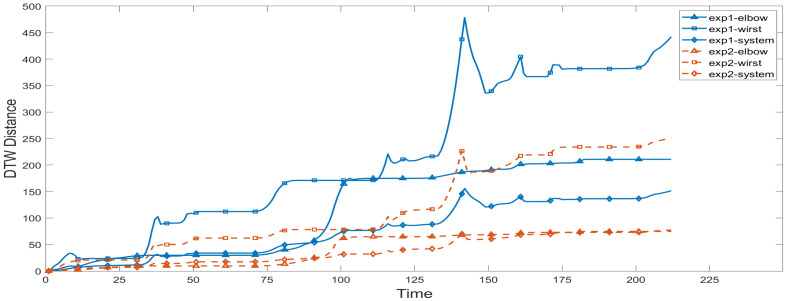
DTW distance in the experiment.

To evaluate the accuracy of the robot’s imitation of human motion trajectories, we randomly generated two human motion trajectories and recorded the joint angles of the robot when the robot imitated the human motion trajectories. It is worth mentioning that the three shoulder joints apply to the conversion from three human DOF to three robot DOF, so that their DTW distances are zeros. According to the range of each degree of freedom, we set $W_{s1}=150,s1\in \left(-30,120\right);$ $W_{s2}=210,s2\in \left(-120,90\right);$ $W_{s3}=40,s3\in \left(-20,20\right);$ $W_{e}=135,e\in \left(0,135\right);$ $W_{w}=180,w\in \left(-180,0\right)$. We calculate the DTW distance of the elbow and the wrist, and finally calculate the DTW distance of the robot system, as shown in Figure 15.

As time elapses, the DTW distance of each joint in each experiment keeps increasing, which is obviously the result of the accumulation of errors generated by the mapping during the experiment. In the curve of the wrist joint, there are obvious special phenomena, and we found the appearance of several sharp points. The reason for this phenomenon is that when the trajectory reaches the cusp, the rotational freedom of the operator’s wrist quickly reaches the limit position, and the bending freedom of the wrist changes greatly, making the action difficult for the robot to imitate. In the subsequent process, the DTW distance is reduced to a normal level because the robot’s actions at the moment are similar to the actions in the subsequent trajectory. According to the characteristics of the DTW algorithm, a better corresponding method will be selected to determine the DTW distance.

During the experiment, the second human motion trajectory we randomly generated had smaller elbow rotation and wrist bending motions than the first one. Therefore, as shown in the figure, the DTW distance of the elbow, wrist, and the whole system in the first experiment is larger than that in the second experiment. What is more, since the bending of the elbow has little effect on the imitation of the robot’s actions, while the rotation of the wrist has a greater influence, in the two experiments, the DTW distance of the wrist is significantly larger than that of the elbow. Since the DTW distance of the shoulder is zero, the DTW distance of the system is generally smaller than the former two.

In general, the DTW-based metrics reveals the following robot imitation characteristics. First, human poses that are difficult for robots to imitate can be identified with a large DTW value and hereby can be avoided at a choreographic stage. Secondly, it is feasible to judge which trajectories can be imitated more accurately and which trajectories are more difficult to imitate among the multiple trajectories in the attainable workspace of the robots.

## 6. Discussion

In this article, we demonstrate a novel teleoperation method that uses lightweight wearable inertial sensors to collect human motion data and map it to the robot. Compared with some existing teleoperation methods, this method adopts first-view mapping, which makes the operator feel more immersive and the robot imitate more accurately. In addition, we propose a DTW trajectory evaluation method, which more accurately describes the similarity between human motion trajectories and robot motion trajectories.

However, our method still has some limitations. In terms of teleoperation methods, firstly, there are differences in the structure of humans and robots. We use a special mapping algorithm, which also means that we have lost part of the data collected by the sensor. This will lead to deviations between robots‘ actions and humans’ actions. In addition, the working space of the robot’s joint angle is limited due to mechanical design, such as the robot’s arm failing to go over the shoulder. Secondly, since the rotation of the human joints is achieved through the rotation of the bones, and the wearable sensor is worn on the surface of the human body, there is a certain angular displacement deviation from the bones. Therefore, when the operator does some special actions, there will be obvious errors. Other factors include accumulated drift error and so on. In terms of DTW trajectory evaluation, although this method describes the similarity between trajectories more accurately and quantitatively, we can only compare it with the method that directly calculates the Euclidean distance. Although this method quantitatively expresses the degree of similarity, the quantitative index can only be used to compare the size with each other to determine the pros and cons, and there is no numerical correspondence.

In future work, we expect to design a more reasonable mapping algorithm, which can reduce the influence of non-isomorphic mapping on the accuracy of robot trajectory simulation through the linkage between joints. Meanwhile, the DTW trajectory evaluation method can be used as an indicator to evaluate whether the mapping algorithm makes the imitation trajectory of the robot more accurate in future research.

## 7. Conclusions

In this article, a human-in-the-loop system for humanoid robots to imitate human motion is proposed, and the metrics of evaluating to what extent the robot motion is similar to that of human are highlighted. The system realizes a real-time simulation and evaluation of humanoid robots through a motion capture system, a fast mapping algorithm, a time series trajectory evaluation method, and multiple visual terminal displays. Under the experiment of a variety of human motion postures, this system has demonstrated good real-time performance and accuracy, and it has also been quantitatively analyzed in terms of the motion similarity evaluation system. This work laid a foundation on improving the robot’s interactive capabilities, especially for human motion imitation.

## Abbreviations

The following abbreviations are used in this manuscript:

HRI	Human–robot interaction
NSFC	The National Natural Science Foundation of China
IK	Inverse kinematics
DOF	Degrees of freedom
BVH	BioVision Hierarchy
IMU	Inertial Measurement Unit
ANP	Axis Neuron Pro
ROS	Robot Operating System
DTW	Dynamic Time Warping
URDF	Unified robot description format

## Appendix A

**Table A1:** Fundamental grammars of Xacro.

Command	Definition	Usage
Property	<xacro:property name=“pi” value=“3.14” />	<⋯ value =“ ${2*pi}”⋯/>
Argument	<xacro:arg name=“use_gui” default=“false”/>	<⋯ use_gui:= true ⋯/>
Macro	<xacro:macro name=“arm" params=“side”/>	<xacro:arm side=“left”/>
Including	<xacro:include filename=“other_file.xacro" />	
